# Examination of ceramic restoration adhesive coverage in cusp-replacement premolar using acoustic emission under fatigue testing

**DOI:** 10.1186/1475-925X-13-165

**Published:** 2014-12-13

**Authors:** Yen-Hsiang Chang, Jin-Jie Yu, Chun-Li Lin

**Affiliations:** Department of General Dentistry, Chang Gung Memorial Hospital, Ding-Hu Road, Kuei-Shan, Tao-Yuan, 333 Taiwan; Department of Biomedical Engineering, National Yang-Ming University, No.155, Sec.2, Linong Street, Taipei, 112 Taiwan

**Keywords:** Cuspal-coverage, Ceramic, Acoustic emission, Fatigue, CAD/CAM

## Abstract

**Background:**

This study investigates CAD/CAM ceramic cusp-replacing restoration resistance with and without buccal cusp replacement under static and dynamic cyclic loads, monitored using the acoustic emission (AE) technique.

**Method:**

The cavity was designed in a typical MODP (mesial-occlusal-distal-palatal) restoration failure shape when the palatal cusp has been lost. Two ceramic restorations [without coverage (WOC) and with (WC) buccal cuspal coverage with 2.0 mm reduction in cuspal height] were prepared to perform the fracture and fatigue tests with normal (200 N) and high (600 N) occlusal forces. The load versus AE signals in the fracture and fatigue tests were recorded to evaluate the restored tooth failure resistance.

**Results:**

The results showed that non-significant differences in load value in the fracture test and the accumulated number of AE signals under normal occlusal force (200 N) in the fatigue test were found between with and without buccal cuspal coverage restorations. The first AE activity occurring for the WOC restoration was lower than that for the WC restoration in the fracture test. The number of AE signals increased with the cyclic load number. The accumulated number of AE signals for the WOC restoration was 187, higher than that (85) for the WC restoration under 600 N in the fatigue test.

**Conclusion:**

The AE technique and fatigue tests employed in this study were used as an assessment tool to evaluate the resistances in large CAD/CAM ceramic restorations. Non-significant differences in the tested fracture loads and accumulated number of AE signals under normal occlusal force (200 N) between different restorations indicated that aggressive treatment (with coverage preparation) in palatal cusp-replacing ceramic premolars require more attention for preserving and protecting the remaining tooth.

## Introduction

With the advances in adhesive methods and ceramic materials, esthetic, metal-free restorations have led to the development of computer design/manufacturing (CAD/CAM) systems for fabricating ceramic inlays, onlays and veneers. CAD/CAM system generated ceramics are currently available that provide a novel means of restoring large cavities in posterior teeth, achieving chair-side design and automated production of all-ceramic monolithic single-unit restorations [1, 2]. While the success of any CAD/CAM restoration is multi-factorial, preparation design and marginal adaptation are vital for successful large replacements [3–8]. Cavity design becomes more complicated and more difficult to manage when preparing for the onlay restoration. The balance between minimizing the risk of tooth fracture and maximizing the repaired tooth function must be carefully engineered. [9–12].

Structural loss with complete cusp fracture in a posterior tooth accompanied by a failed Class II MOD (mesial-occlusal-distal) restoration is a common phenomenon in dental practice [9, 13, 14]. When a bonded cusp-replacing adhesive restoration depends only on an adhesive system without any accessory retention or resistance in the cavity preparation, the retention strength is often doubtful, as the restored tooth undergoes repeated loads in the oral environment [15]. Kuijs et al. stated that an additional cervical shoulder preparation does not improve the fracture strength of cusp-replacing direct resin composite restorations as long as some retentive form is present [16]. Fennis et al. reported that palatal cuspal coverage increased the fatigue resistance of a direct resin composite restoration [10]. However, these two studies focused only on resin composite restorations and not on CAD/CAM ceramic materials. Chang et al. used FEA and indicated that buccal cusp reduction of at least 1.5 mm presented more favorable biomechanical performance when cuspal-coverage treatment is considered [3]. Unfortunately, the result of this static numerical study requires further evaluation using insight into the in-vitro fatigue tests to provide more realistic consultation.

The acoustic emission (AE) technique is a non-destructive technique using a sound wave produced by energy released from an externally stimulated material [17]. Locations with flaws under load develop high stress energies and some of this energy is released in the form of a pressure wave or sound that occurs long before the specimen fails catastrophically. This technique can be used to detect the onset and failure progression in structures [17–19]. Combined with conventional static fracture test machines, AE can identify failure initiation, the initial damage site, damage propagation and catastrophic failure of the material and help elucidate the complex failure mechanism [17, 20–26]. However, the AE technique has rarely been employed in dental ceramic fracture testing under cyclic loads. Our previous study successfully combined the AE technique with fatigue shear testing to investigate micro-crack growth and damage in ceramic/dentin adhesive interfaces. The result found that the cumulative number of AE hits increased with a lower load level in cyclic load tests before fracture [27].

Accordingly, this study applied the AE technique to monitor the resistance in CAD/CAM ceramic restorations with and without buccal cusp replacement under static and dynamic cyclic loads. AE signal results at different cyclic load stages were obtained to understand the biomechanical response of premolar ceramic cuspal-replacing restorations.

## Materials and methods

### Sample preparation

**Figure 1:**
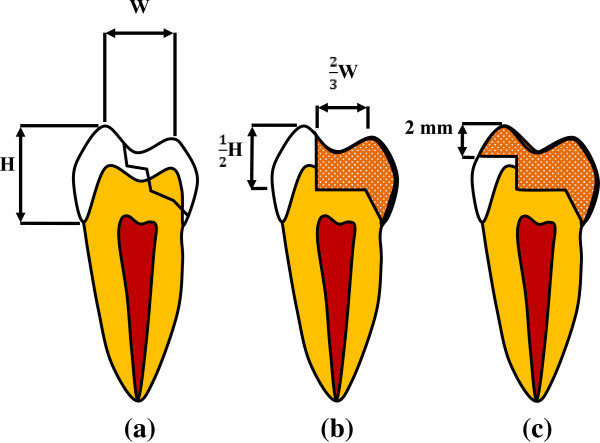
**Cavities definition. (a)** Intact premolar with typical MODP (mesial-occlusal-distal- palatal); i.e., loss of a functional cusp with a 45-degree bevel surface at the cervical margin; **(b)** Maxillary premolar with palatal cusp fracture without buccal coverage preparation (WOC); **(c)** Maxillary premolar with palatal cusp fracture with buccal cusp reduced in 2 mm height (WC).

Eighteen freshly extracted intact maxillary second premolars were stripped of ligaments and stored at 18°C in normal saline and randomly assigned to two groups of 9 teeth each. Teeth with similar size and shape were selected using root length and crown dimensions after measuring the buccolingual and mesiodistal widths at the cement-enamel junction (CEJ) in millimeters, allowing for a maximum deviation of 20% from the mean. All teeth were embedded from the root to 1-mm below the CEJ into an epoxy resin block.The cavity was designed in a typical MODP (mesial-occlusal-distal- palatal); i.e., loss of a functional cusp with a 45-degree bevel surface at the cervical margin. The pulpal wall was designed at half the distance between the buccal cusp tip and the CEJ (H), and the isthmus width was two-thirds of the intercuspal width (W) (Figure 1a and b). Two cavity types with 9 teeth in each group were prepared with palatal cusp-replacing ceramic restorations without (WOC) and with (WC) buccal cuspal coverage (Figure 1b and c). The cavity preparation with buccal cuspal coverage was reduced in cuspal height by 2.0 mm (Figure 1c). The specimen teeth preparation all according to follow steps: Shofu 311 cylinder diamond bur was used to prepare a typical MOD cavity (also used for preparing groups with loss of the buccal cusps teeth) and 45 degree bevel surface was prepared along MOD cavity palatal axiopulpal line angle (simulate a palatal cusp loss on a premolar) by Shofu 211 cylinder diamond bur.

Both the WOC and WC onlay ceramic restorations were designed using the CEREC 3D CAD/CAM unit (Version 3, BlueCam, Sirona, Bensheim, Germany) and machined from ProCAD Lucite-reinforced ceramic blocks (200, I14; Ivoclar Vivadent). Before insertion, the bonding surfaces of the ceramic onlays were etched with hydrofluoric acid (Ultradent Porcelain Etch, 6%; Ultradent Products, South Jordan, UT, USA) for 90 seconds, then dried for 30 seconds in oil-free air. A silane-coupling agent (Monobond S; Ivoclar Vivadent) was applied and allowed to dry for 1 minute. An etch-and-rinse Variolink II adhesive system was applied to bond the tooth structure and ceramic. The exposed tooth structure was acid etched with 35% phosphoric acid gel 15 sec and air-dried. Heliobond was uniformly applied to the tooth surface. Onlays were seated with light finger pressure and excess luting material was removed. A light-polymerizing unit (Bluephase G2, Ivoclar Vivadent, Liechtenstein) was held on the buccal, mesial, lingual, distal and occlusal surfaces for 40 seconds to 1 minute for each surface. The curing power was 1200 mW/cm^2^.

### Fracture and fatigue tests

**Figure 2:**
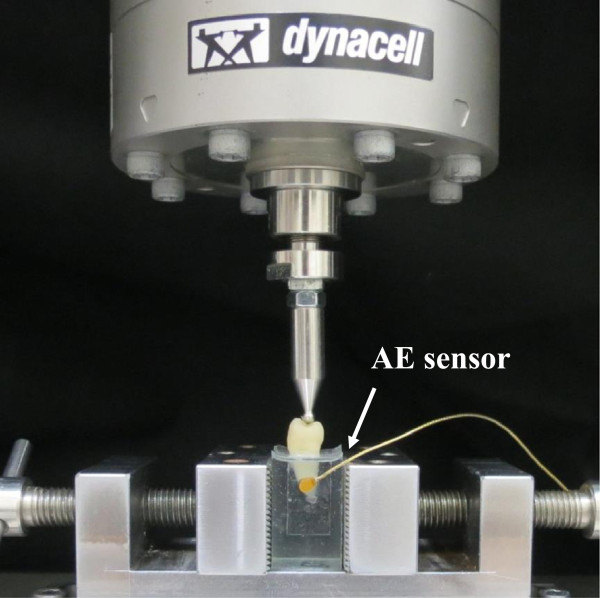
**One of the tested restored tooth samples included a stainless steel sphere in contact with the buccal and lingual cusps for axial load and an AE signal wide band transducer glued with resin to the sample embedded in a resin block.**

Three samples from the WOC and WC groups were randomly selected and mounted vertically to the long tooth axis for fixation in a universal testing machine (E3000, Instron, Canton, MA, USA). The test system was designed to drive a static compression force onto the tooth with a 4-mm steel sphere contacting the buccal and lingual cusps for axial loading (Figure 2). The crosshead speed was set at 0.05 mm/s until a fracture occurred. The fracture load value was recorded.

The fatigue test cyclic loads were carried out by applying 200 N and 600 N onto the tooth with a 4-mm steel sphere contacting the buccal and lingual cusps to simulate the occlusal forces, respectively [28, 29]. The R value (Fmax/Fmin) was set at 10. The test frequency was set at 4 Hz because the human mastication frequency was found to be 1 Hz to 4 Hz from the literature [30, 31]. Three samples each from the WOC and WC groups were tested at each cyclic load (200 N and 600 N). The number of cycles at each load was set at 10^5^ because this number simulated chewing and swallowing for one half year [32, 33]. A preload of 5 N was applied first on the tooth to test the stability between steel sphere and tooth before performing the static/dynamic compressive test.

### AE analysis

An AE signal wide band transducer (Broadband sensor S9225, Physical Acoustic Corporation (PAC) Princeton Junction, NJ, USA) was glued with resin (Triad Gel, Dentsply, York, PA, USA) to the resin block in which the sample was embedded for the static compressive and cyclic load tests (Figure 2). Signals detected by the transducer were passed through 40 dB gain preamplifiers with a band pass of 100 k ~ 2 MHz (Model 2/4/6, PAC) [17, 20, 34]. The AE signals were recorded during the load period. The load versus AE hit in the fracture test and number of cycles versus AE hits in the cyclic load test (every 5000 of cycle load) for each sample were recorded to evaluate how the restored tooth failure process differed between static and fatigue tests. AE signal mean and standard deviations accumulated at each recorded time were computed. All data were statistically analyzed using the t-test method (α =0.05).

## Results

**Figure 3:**
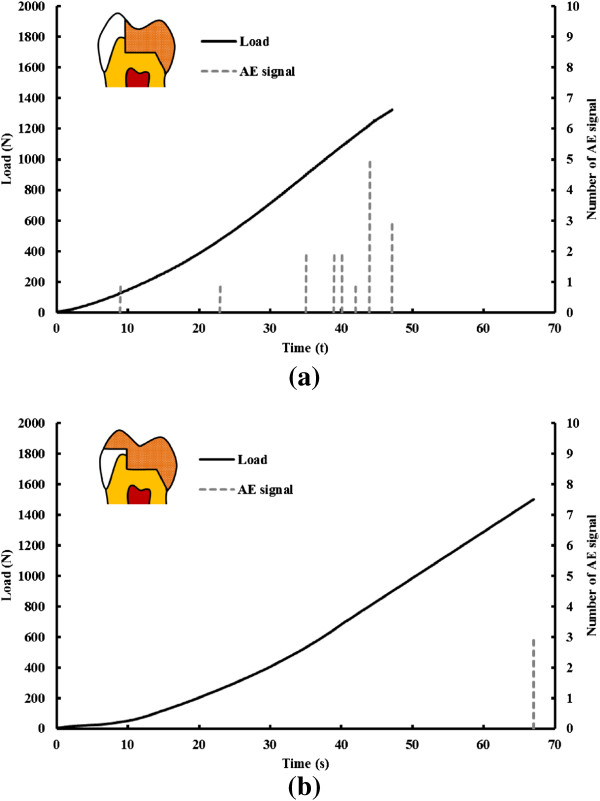
**Typical example of AE signals in load/loading time graphs in fracture test. (a)** WOC restoration and **(b)** WC restoration.

**Figure 4:**
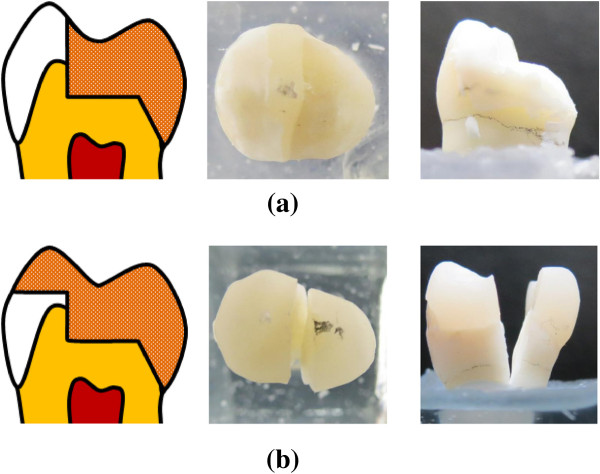
**Fracture modes of the (a) WOC and (b) WC restored teeth after fracture tests.**

The t-test result in the fracture test displayed non-significant differences (p >0.05) between the tested fracture loads for the mean WOC and WC restoration values (Table 1). However, significant differences (p < 0.05) were found in the accumulated number of AE signals between these two restorations. The mean number of 15 for the WOC restoration was higher than 4 for the WC restoration. Generally, the tested loads and time required for the first AE activity for the WOC restoration in all samples were lower than that for the WC restoration. Test sample 2 in the WOC and WC restorations was selected on behalf of all test samples to show the typical load versus AE hit diagram in the fracture test. The result showed that tested loads (130 N) and time (9 s) required for the first AE activity for the WOC restoration was lower than that for the WC (1501 N and 67 s) restoration (Figure 3). Different fracture modes in the restored teeth after fracture tests are shown in Figure 4.

**Table 1:** **Fracture load and accumulated number of AE signal in static fracture test**

Restoration design	Fracture load (N)	P value	Accumulated number of AE signal (hit)	P value
	(Mean ± SD)		(Mean ± SD)	
WOC	1427 ± 309	0.322	15 ± 2	*0.018
WC	1559 ± 337		4 ± 2	

*significant difference (p < 0.05).

**Figure 5:**
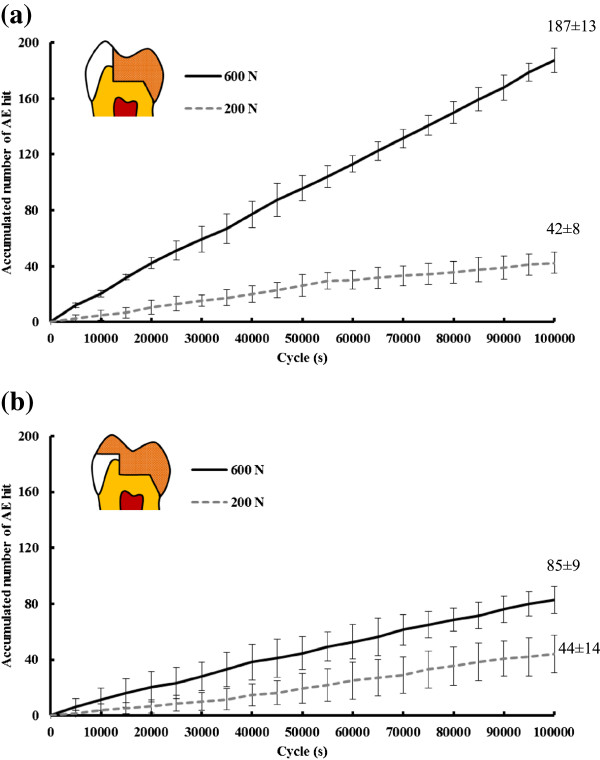
**Accumulated number of AE signals versus number of cyclic load during normal (200 N) and high (600 N) occlusal forces fatigue testing for: (a) WOC restoration and (b) WC restoration.**

No visible crack or damage was found in all samples after 10^5^ cycle loads with 200 N or 600 N according to the fatigue test results. The number of AE signals increased with the cycle load number and the accumulated number of AE signals under 600 N were higher than that under 200 N. After 10^5^ cyclic loads non-significant differences (p > 0.05) in the accumulated number of AE signals were found between the WOC (42 ± 8) and WC (44 ± 14) restorations under the 200 N condition. Conversely, the corresponding mean value 187 (±13) for the WOC restoration was significantly higher than that of 85 (±9) for the WC restoration under the 600 N condition (Figure 5).

## Discussion

Developed over two decades ago, the challenge of high performance ceramic and shaping it quickly and predictably to fit the defect in a damaged tooth has been the impetus behind advances in computer controlled machining in dentistry [4, 6, 20]. The CAD/CAM system has proven capable of restoring a large cavity chair side without the need for an impression or laboratory assistance [4–6]. However, the extensive cusp-replacing restoration fabricated without any reinforcement has doubtful structural stability and adhesive bond strength in long-term use. With special risk of cuspal fracture in an MODP restoration in premolars, Hannig et al. advocated that research should focus on in-vivo and in vitro studies evaluating ceramic restorations for reinforcement with and without cuspal (partial or complete) coverage [35]. In 2004 Fennis et al. reported that palatal cuspal coverage increased the fatigue resistance of direct resin composite restorations. However, the direct resin composite cusp-replacement fracture mode might be unsuitable for restorations with CAD/CAM ceramic restorations [10]. Although our previous study indicated that cusp-replacement treatment was recommended to prevent palatal cusp fracture in extensively restored MODP premolar restorations. Reduction of the buccal cusp by at least 1.5 mm was seen to reduce stress. However, the clinical significance of the results from static FE analysis is occasionally questionable because a monotonic load does not accurately represent a clinical situation in which repetitive fatigue loading is characteristic [3].

Repeated load fatigue has been recognized as an important concept rather than a catastrophic event, inducing ceramic restoration failure. The AE technique has also been employed in dynamic fatigue testing in orthopaedic/dental materials. Roques et al. [18] used AE to assess bone cement failure using four point bending fatigue tests. Their results indicated that the AE technique can be used as a pre-clinical assessment tool for the integrity of cemented load bearing implants. Our previous study also used the AE technique to investigate micro-crack growth and damage in the ceramic/dentin adhesive interface under fatigue shear testing and found that the cumulative number of AE hits increased with a lower load level in cyclic load tests before fracture [27]. Therefore, this study attempted to combine the AE technique to monitor the failure process in CAD/CAM ceramic restored tooth with and without buccal cusp replacement under dynamic loads.

A small sample size with wide variability in the dimensions and mechanical properties of the teeth might cause the result to be judged with the “banal” argument. A study on a small sample is quite tempting and if the sample size is too small, the power of the results may be low to the point of unreliably. However, the importance of sample size calculation cannot be overemphasized because this study can be conducted for various objectives. In order to reduce the variability in the dimensions of the collected teeth, teeth with similar size and shape were selected using the root length and crown dimensions after measuring the buccolingual and mesiodistal widths at the cement-enamel junction (CEJ) in millimeters, and allowing for a maximum deviation of 20% from the mean. However, the age of the patients with wide variability of the mechanical properties of the teeth was our limitation in this study.

Non-significant differences (p > 0.05) between the tested fracture loads were found for the mean WOC and WC restoration values (Table 1). High standard deviations due to small size samples in the fracture test or unreasonable assumption (PDL non-linear material property, ill-condition element, loading and boundary conditions) in previous simulated FE models might be factors that caused the experimental result to not be consistent with previous FE studies. However, the AE signal was accompanied with final fracture, exhibiting the mechanism was prone to brittle fracture caused by catastrophic loading in the WC restored tooth (Figure 3(b)). The fracture type found in the WC restored tooth was from the central fossa to the deep root region and indicated that this catastrophic failure cannot be repaired again when fracture has occurred (Figure 4(b)). Conversely, the first AE signal relative to the received WOC restored tooth load was lower than the final fracture magnitude. The accumulated number of AE signals exceeded more than 13 in causing the WOC restored tooth to fracture (Figure 3(b)). This phenomenon revealed that micro-cracks might be present in the tooth structure, cement layer or ceramic within the WOC restored tooth to propagate and eventually coalescence into larger cracks leading to failure [36]. The fracture in the WOC restored tooth was only found in the restored ceramic (Figure 4(a)).

The situation regarding the number of occlusal contacts that occur in vivo, Delong et al. addressed that chewing tooth contacts equal 240000 in one year [32]. Ruse et al. [37] pointed out that chewing and swallowing contacts equal 1800 per day. These numbers translate into 10^5^ in 2–5 months. Owing to the constrained time available for collecting data, a 10^5^ cyclic load was used as the relative number to simulate clinical chewing.The fracture test with fatigue test under 200 N indicated that significant differences in the accumulated number of AE signals cannot be found between WOC and WC restorations (Figure 5). This result pointed out that treatment in palatal cusp-replacing ceramic premolar restoration without (WOC) buccal cuspal coverage might withstand normal occlusal force for at least half a year. However, the accumulated number of AE signals for the WOC restoration was significantly higher than that for the WC restoration under the 600 N condition (Figure 5). High accumulated number of AE signals addressed the possibility of micro leakage in the restored tooth, in the tooth structure, cement layer or ceramic needed further consideration for long term use. The result also implied that people with para-function, heavy occlusal, bruxism may need treatment with buccal cuspal coverage to resist great occlusal force with high frequency. The aim of this study was to investigate the proper restorative technique for premolars with large functional cusp fractures. Due to the advancements in adhesive dentistry, onlay restoration has become an excellent conservation treatment option for these teeth. Preserving and protecting the remaining tooth structure should be the primary goal for all restorative systems. A fractured restoration can be remade, but a fractured tooth may become non-restorable. Therefore, emphases on protecting the remaining tooth structure with care in aggressive treatment (with coverage preparation) are needed.

This *in vitro* experimental study was designed to understand the relationship between the accumulated number of AE signals and fracture (load) resistance in preparation cavity designs for cusp-replacing restorations. Some clinical situations cannot be fully represented in this study and need to be assumed, such as load direction condition, cement thickness and the absence of periodontal ligaments (PDL) in the test samples. The resulting axial force crossing the central position (fracture or dynamic 200 N/600 N) force assumed in this study was not realistic and only approximated the complex balance between the masticatory forces and their reactions. However, the axial contact force transferred to the restored tooth may be resolved in tensile, shear and compressive forces on the adhesive interface, restorative ceramic and remaining tooth, except for near the load areas. Although there was an in-vitro test indicated for ceramic systems, there was no correlation between the cement thickness and micro-leakage either in the enamel or dentin [38]. Minimizing the gap between the prepared cavity and the restorative material should still be the goal for any restorative technique. Despite PDL not being simulated in our study, PDL either behaves as a rigid or non-rigid element, it is far away from the crown region and the stress distribution in the crown restoration may be assumed independent of the root region. This statement can be explained by the Saint-Venant principle [39].

## Conclusion

Within the limitations of this *in vitro* study, the non-significant differences in results in the tested fracture loads and accumulated number of AE signals with normal occlusal force under fatigue testing implied that aggressive treatment (with buccal coverage preparation) requires more care in preserving and protecting the remaining tooth structure for palatal cusp-replacing ceramic premolar treatment. However, aggressive buccal coverage preparation for a palatal cusp-replacing ceramic premolar can be considered as the treatment type to gain stronger resistance from the restored tooth when it frequently receives high occlusal force.
